# The Defensive Interactions of Prominent Infectious Protozoan Parasites: The Host’s Complement System

**DOI:** 10.3390/biom12111564

**Published:** 2022-10-26

**Authors:** Sajad Rashidi, Reza Mansouri, Mohammad Ali-Hassanzadeh, Antonio Muro, Paul Nguewa, Raúl Manzano-Román

**Affiliations:** 1 Molecular and Medicine Research Center, Khomein University of Medical Sciences, Khomein 38811, Iran; 2 Department of Medical Laboratory Sciences, Khomein University of Medical Sciences, Khomein 38811, Iran; 3 Department of Immunology, Faculty of Medicine, Shahid Sadoughi University of Medical Sciences and Health Services, Yazd 8915173143, Iran; 4 Department of Immunology, School of Medicine, Jiroft University of Medical Sciences, Jiroft 7861615765, Iran; 5 Infectious and Tropical Diseases Group (e-INTRO), Institute of Biomedical Research of Salamanca-Research Center for Tropical Diseases at the University of Salamanca (IBSAL-CIETUS), Faculty of Pharmacy, University of Salamanca, 37008 Salamanca, Spain; 6 Department of Microbiology and Parasitology, ISTUN Institute of Tropical Health, IdiSNA (Navarra Institute for Health Research), University of Navarra, C/Irunlarrea 1, 31008 Pamplona, Spain

**Keywords:** protozoan parasites, complement system, complement regulatory proteins, complement evasion molecular interactions

## Abstract

The complement system exerts crucial functions both in innate immune responses and adaptive humoral immunity. This pivotal system plays a major role dealing with pathogen invasions including protozoan parasites. Different pathogens including parasites have developed sophisticated strategies to defend themselves against complement killing. Some of these strategies include the employment, mimicking or inhibition of host’s complement regulatory proteins, leading to complement evasion. Therefore, parasites are proven to use the manipulation of the complement system to assist them during infection and persistence. Herein, we attempt to study the interaction´s mechanisms of some prominent infectious protozoan parasites including *Plasmodium*, *Toxoplasma*, *Trypanosoma*, and *Leishmania* dealing with the complement system. Moreover, several crucial proteins that are expressed, recruited or hijacked by parasites and are involved in the modulation of the host´s complement system are selected and their role for efficient complement killing or lysis evasion is discussed. In addition, parasite’s complement regulatory proteins appear as plausible therapeutic and vaccine targets in protozoan parasitic infections. Accordingly, we also suggest some perspectives and insights useful in guiding future investigations.

## 1. Introduction

Intracellular protozoan parasites especially *Leishmania*, *Toxoplasma*, *Plasmodium* and *Trypanosomes* manipulate the infected-host cell homeostasis to favor parasite replication and survival and to induce pathogenesis [1]. These parasites employ different strategies to circumvent the cell signaling cascades and subsequent host cell immune responses to promote a favorable infectious environment [2,3]. In this regard, immune modulation is one of the most prominent strategies used by intracellular protozoan parasites [4,5]. The most likely mechanisms that exert major function in parasite-immune evasion, pathogenesis and persistence are mimicking, hijacking and the inhibition of different molecules of the host´s immune responses [5]. 

**Figure 1:**
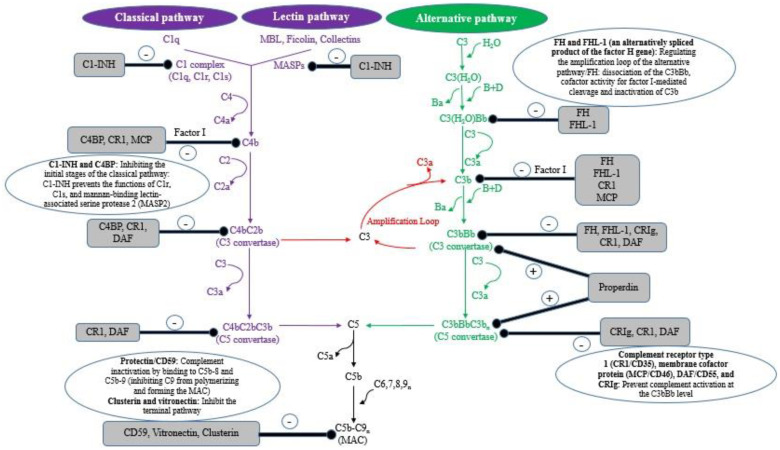
The activation of complement system: (a) the classical pathway is induced by the recognition of antibody-antigen complexes on the pathogens surfaces through (by interacting with) complement component C1q, then cleaving C2 and C4 to produce C2a and C4b; these complement components bind to the pathogens surface to compose the C3 convertase C4b2a. (b) The lectin pathway in triggered by binding the mannose-binding lectin (MBL) or ficolin to mannan or glycosylated biomolecules, respectively (on the pathogens surface). The interaction of cysteine proteases with these biomolecules drive to the cleavage of C2 and C4 and thus generate the C3 convertase C4b2a. (c) The alternative pathway is initiated by spontaneously hydrolyzing C3b or C3b obtained from the other pathways, interacting with factor B, which is cleaved into Bb through factor D, and consequently composing the C3 convertase C3bBb [6,7].

The complement system, as part of the innate immunity, has important functions, such as cell opsonization during phagocytosis (through complement components C1q-, C3b-, and iC3b), immune cell recruitment, inflammation induction (through the release of C3a and C5a) and during the formation of the Membrane Attack Complex (MAC) leading to pathogens lysis during infections. The complement includes the classical (CP), alternative (AP), and lectin pathways (LP) (Figure 1). C3 convertase originated from all pathways interact with the newly cleaved C3b, composing a C5 convertase (cleaving C5 into C5b). C5b interacts with C6-C9, thus forming the MAC and subsequently pathogen killing. All complement pathways are different in their initial steps, however, all of them are common in producing a C3 convertase, a C5 convertase, the formation of the MAC and finally inducing pathogen lysis. The complement is controlled and regulated through different mechanisms, including by fluid phase regulators (factor H (FH), factor H-like protein 1 (FHL-1), C1-inhibitor (C1-INH), and C4b-binding protein (C4BP)), and cell membrane regulators (complement receptor 1 (CR1)/CD35, decay-accelerating factor (DAF)/CD55, membrane cofactor protein (MCP/CD46), protectin/CD59, and complement receptor of immunoglobulin (CRIg)). Those pathways allow for the restriction of overactivation of the complement or inappropriate complement activation and also to control host cell damage (Figure 1) [6,7,8].

Pathogens can efficiently manage the overactivation of the complement system by targeting complement regulatory mechanisms [9,10]. In this sense, pathogens interact with or evade complement through various approaches including (i) the inhibition of MAC formation, (ii) the expression of proteins that mimic or hijack host surface and fluid-bound complement regulators, (iii) the secretion of complement inhibitory proteins (such as proteases), (iv) the recruitment of complement opsonization factors to enhance intracellular invasion, (v) the interference with anaphylatoxin signaling, and (vi) the modulation of the convertase enzyme formation or stability. Surprisingly, some pathogens may apply simultaneously redundant or different evasion strategies to induce an effective and efficient complement evasion [8,11].

**Figure 2:**
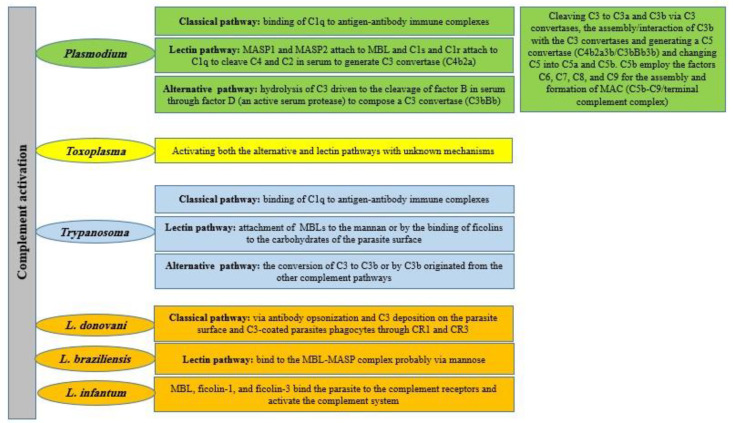
The complement system activation in the most prominent and challenging protozoan parasitic infections [6,16,17,18].

During protozoan parasitic infections, the complement system might also be activated by a proteolytic cascade that consequently drives to the opsonization and lysis of invading parasites (Figure 2) [12,13]. It mostly exerts its function through the formation of the MAC and the development of an inflammatory reaction on the parasite´s surface. However, parasites have developed deceptive tactics leading to the inhibition of complement functions and the MAC formation mainly through the mimicking or hijacking of the employment of host complement regulatory proteins, the expression of orthologs of Regulators of Complement Activation (RCA) on the parasite´s surface and also by the expression of redundant parasite-encoded proteins targeting different complement components [14]. Accordingly, this review highlights some of the most outstanding proteins and the main molecular mechanisms potentially acting as complement evasion strategies leading to successful protozoan parasites survival and persistence, thus potentially serving as targets to manage mainly devastating neglected tropical diseases [15]. 

## 2. Parasite’s Complement Regulatory and Evasion Molecules

### 2.1. Plasmodium spp.

These are the causative agents of malaria, an acute febrile disease with 241 million cases and 627,000 deaths worldwide in 2020. *Plasmodium* spp. belong to the apicomplexan parasites with a complex life cycle involving two hosts, an invertebrate, the *Anopheles* mosquito acting as its biological vector and a vertebrate, such as humans [19,20]. It has been found that the complement is involved in all life cycle-stages of the *Plasmodium* parasites, including the pre-erythrocytic-, erythrocytic-, and sexual-stages during immunity against malaria [7,21,22]. 

The antibody response in a complement-dependent manner could be an important contributor to natural acquired immunity against protozoan parasites [7,16,21]. For instance, the direct inhibitory effect of antibodies against the sporozoites (CSP), merozoites (MSP119), and sexual stages of *Plasmodium falciparum* (Pfs230) surface proteins can be efficiently favored due to the complement activation. In this sense, the attachment of C1q component to IgG-opsonized sporozoites and merozoites was correlated to a protective immunity in malaria patients [16]. Antibody-mediated complement-dependent (Ab-C’) inhibition is relevant to prevent merozoite invasion of Red Blood Cells (RBCs) during blood-stage malaria infections. C1q fixation has been represented as the main mediator of Ab-C’ inhibition through binding to the *Plasmodium* surface proteins MSP-1 and MSP-2 [23,24]. However, blood infected erythrocytes are resistant to antibody-complement mediated lysis compared to the other parasitic life cycle stages. This might due to several intrinsic aspects of infected erythrocytes, including the expression of complement regulatory proteins on infected RBCs, the lower number of antigenic binding sites for antibodies, and the orientation or variation in antigenic targets on the parasite´s surface to inhibit antibody attachment and consequently complement activation [16]. 

By another way, the antibody-dependent complement action can play a major role in parasite phagocytosis or opsonophagocytosis by the attachment of antibodies to *Plasmodium* parasites during the infection [13]. However, these could also be helpful to favor parasite replication and survival at various life stages, including the erythrocyte infection and for rosette formation which inhibits complement activation and promotes infection [25,26].

Free merozoites can bind both FH and FHL-1 to their surfaces (most probably through merozoite protein Pf92 and binding to the CCP domains 5-6 of FH and FHL-1), inactivating C3b and increasing parasite survival during the erythrocytic replication phase [27,28,29]. Therefore, mimicking or hijacking the FH and FHL-1 factors might also be a possible evasion strategy of these parasite life cycle-stages. FH (formerly known as b1H), as a fluid phase complement regulatory protein belongs to a family of proteins including FH, FHL-1 and Factor-H related proteins (FHRs). Surprisingly, increasing evidence has shown that FHRs play opposite functions compared to factor H and FHL-1; this is the direct enhancement of complement activation in competition with the regulators FH and FHL-1 [30]. FH can negatively regulate the AP pathway and it has been also recognized as the regulator of the CP pathway being thus the main target of different pathogens for AP evasion [8]. Moreover, FHR-1 may compete to bind to plasmodial FH receptors and impair FH regulatory activity and C3b inactivation on the parasite surface. These data suggest that FHR-1 can act as a protective factor in host’s immunity by counteracting FH-mediated parasite complement evasion [31]. By expressing other merozoite surface biomarkers, such as C1-INH and CR1, acting as a soluble regulator of the complement activation; these parasites can negatively modulate both classical and lectin pathways by inhibiting C1s, C1r, mucin-associated surface protein-1(MASP-1) and MASP-2 (the activator proteases of the complement system) [14]. PfMSP3.1 and *P. falciparum* glycosylphosphatidylinositol (PfGPI) probably play a major function in merozoite binding to the C1-INH. This attachment leads to the limitation of classical pathway proteases and consequently the inactivation of downstream complement cascade [27,32,33]. Interestingly, the decreased expression level of CR1 on the surface of uninfected erythrocytes (increasing erythrocyte susceptibility to macrophage clearance) is most probably essential for the clearance of these erythrocytes in malarial anemia caused by *P. falciparum* and *P. vivax*. The high expression level of CRs on infected compared to uninfected erythrocytes indicated that parasite invasion of erythrocytes through CRP receptor pathways is related to parasite evasion mechanisms from complement-mediated clearance [34,35].

On the other hand, *P. falciparum* can also differentially express on their surface orthologs or receptors of host soluble complement components. These biomolecules can also act as targets for antibodies (antibody-mediated complement lysis). Specially, sporozoites express different proteins on their surfaces to interfere with complement and neutralize the activity of this system. DAF triggers the decay of C3 and C5 convertases in association with C3b and C4b deposited on the cell membrane [14]. Evidence has shown that the expression of parasite-like DAF in salivary gland sporozoites induces their resistance to lysis by the complement [36]. 

In the asexual erythrocytic stages, the antigenic variation (especially regarding *P. falciparum* erythrocyte membrane protein 1 (PfEMP1)) on the *P. falciparum*-infected erythrocytes surface might mediate the limitation of complement fixation and cell lysis. Other mechanisms including expressing host orthologs or capturing the host cell-intrinsic complement regulator CD59 may also allow the parasites to prevent the formation of MAC on infected RBCs mediated by parasite mannosyltransferase (PfPIG-M) [7,37,38]. In addition, blocking C1q-binding site on IgM and IgG might be considered as *Plasmodium*’s strategies to evade complement [7,38]. Finally, *Plasmodium* parasites with an unknown mechanism attach to the host cells plasminogen and mediate its conversion to plasmin during the intraerythrocytic stage, leading to the inactivation of C3b and complement evasion [39]. Since plasminogen and related proteins (plasminogen-binding proteins) are involved in parasites invasion and migration through the tissues of the infected host [40], more investigations may decipher information regarding the role of such proteins in parasite evasion from complement system. 

Regarding the mosquito stages, *P. falciparum* gametes recruit, express or mimic FH and FHL-1 from the ingested human blood to their surfaces through different mechanisms including probably the attachment to parasite protein glideosome-associated *protein* 50 (GAP50) to inactivate C3b [41]. Moreover, the covering of the gametes of *Plasmodium* by cellular debris of infected-RBCs during gametogenesis can further augment the gametes immune evasion [42]. Surprisingly, the mosquitoes midgut cells can also capture and employ FH via specific receptors inactivating C3b and inhibiting the C3bBb enzymes formation to protect themselves from complement lysis [43]. Interestingly, evidence has revealed that 6-CYS protein Pfs47 can facilitate *Plasmodium* parasites transmission in mosquitos by its ability to regulate the insect complement-like immune system [44]. Pfs47 probably disrupts c-Jun N-terminal kinases (JNKs) pathway-mediated apoptosis and inhibits nitration of ookinete, which are essential to activate the complement-like system [45,46]. In addition, it has been recently clarified that some proteins, including PIMMS43 (*Plasmodium* Infection of the Mosquito Midgut Screen 43) and CSP are expressed in *Plasmodium* oocysts to induce parasite evasion of the mosquito complement-like response [47,48]. All these mechanisms can further highlight that *Plasmodium* parasites can evade the complement system in different life cycle stages.

### 2.2. Toxoplasma spp.

*Toxoplasma gondii* is the causative agent of toxoplasmosis, a prevalent world zoonotic infectious disease, with seropositivity rates of 10% to more than 90% depending on the geographical area. Toxoplasmosis is mostly asymptomatic, however, in immunosuppressed patients it appears as a symptomatic disease. In pregnant women with an acute form of the infection, it could be fatal for the fetus. There are different evolutionary forms in *T. gondii*-life cycle being the felines considered as definitive hosts and humans as intermediate hosts [49].

*T. gondii* activates both the AP and LP pathways and actively resists complement-mediated lysis in non-immune human serum through inactivating C3b. *Toxoplasma* hijacks both CP and LP regulator C4b-binding proteins (C4BP) and AP regulator FH to the parasite surface to inhibit bound C3b-iC3b and C3dg and restrict formation of the C5b-9 attack complex. It seems that modulating the alternative pathway by hijacking FH is more crucial for parasite resistance. *Toxoplasma* parasite is capable of binding heparan sulfated proteoglycans (SPGs) and sialic acid through microneme proteins 1 and 4 (MIC1, MIC4) and surface antigen-related sequence 57 (SRS57) to hijack or recruit FH and C4BP [12,50]. In general, evidence has shown that *Toxoplasma* significantly modulates the form and level of C3b on its surface to recruit complement and consequently modulate adaptive immunity to increase virulence. For instance, it seems that the surface of *Toxoplasma* is covered by iC3b and C3dg. The covalent binding of surface antigens with C3dg can affect B cell immunity. The opsonization with iC3b can also influence the phagocytosis of this parasite leading to complement evasion [50].

*T. gondii* and *Plasmodium* spp. are obligate intracellular protozoan parasites that can significantly lead to the dysfunction and injury in the brain due to the interactions between these protozoa and complement system (Table 1) [51]. It seems that the overactivation or overexpression of host’s complement components by *Plasmodium* parasites (or even on the parasite surface) participates in cerebral malaria (CM) pathogenesis. Thus, the inhibition of complement system at the level or downstream of C5 activation or CR1 (CD35) might be suggested as a therapeutic strategy against CM. Although complement-dependent clearance is required during the initial stage of *Toxopolasma* spp. infection, these parasites can induce persistent complement activation in the central nervous system (CNS) in chronic stage [51].

The initial passage of *Toxoplasma* parasite to the CNS occurs across cortical capillaries. However, the integrity of the microvascular blood–brain barrier (BBB) decreases parasite transmission, which consequently is further augmented by the inflammatory response [52]. Several evidences have shown that complement and coagulation cascades pathway and tight junction pathway are correlated with the host immune system and the brain intrusion of parasite in *Toxoplasma*-infected mice, respectively. *Toxoplasma* parasite can significatively induce and upregulate the C3 and C1q and disrupt the tight junction of the BBB in the CNS thus facilitating the parasite penetration to the brain tissue via the intercellular space [53]. Globally, the increase of C4b, C3 and C1q during toxoplasmosis infection induces nerve cell disruption including synaptic loss and other plausible neurodegeneration in addition to their roles for parasite entrance [54].

Microglia and astrocytes cells and their crosstalk regulate homeostasis in the normal brain tissue and *Toxoplasma* parasites can induce different physiological effects on the glia and astrocytes most probably through altering immune system responses [55,56,57,58]. Since these cells are an important part of the CNS, their activity modulation or dysfunction could drive to the neuroinflammation and various brain disorders [59,60]. There is growing information suggesting crucial functions of complement components in microglia-mediated neuropathological disorders [61,62]. Microglia cells mediate and facilitate the *Toxoplasma* parasite dissemination in the CNS and also participate to the induction of complement activity in different brain regions [63]. Evidences have shown that alternative complement components and anaphylatoxin receptors signals could be upregulated in the *Toxoplasma*-infected brain parenchyma through parasite-microglia interactions. *Toxoplasma* parasites can directly upregulate the complement factor B (CFB), complement component factor properdin (CFP), C3, and C5aR1 in glial cells. Since the C5a/C5aR1 axis increases interleukin 12 (IL-12) induction in splenic dendritic cells and subsequent inducible nitric oxide synthase (iNOS) expression in the brain [64], the continued overexpression of C3aR and C5aR1 in the infected brains could be probably correlated with the defense against *Toxoplasma* infection-induced damages in the CNS [63].

**Table 1:** Interactions between *Toxoplasma*/*Plasmodium* parasites and complement system in infected brain tissue.

Interaction Mechanisms with the Tissue (Brain)	References
*Plasmodium* spp.	
Upregulation of C1q and C5 in CM patients	[65,66]
C5a up-regulation (C5aR deficiency: increasing productivity against CM) C5aR probably mediates persistent neurocognitive deficits	[67,68]
Inducing innate immune responses (including the complement system) and associated demyelination (severity of CM)	[65]
C5 deficiency and C5aR blockade were protective against CM (protection of C5 deficient mice against CM is mediated via the inhibition of MAC, not through C5a-induced inflammation)	[69,70]
C5 plays a role in malaria-induced seizures	[71]
T cell-deficiency is protective against CM (which was correlated by decreased complement activation)	[72]
Dysregulated C5aR signaling participates in the pathogenesis	[67]
C9 deposition throughout the cortex of cerebral malaria (CM progression)	[70]
*Toxoplasma*	
C3 and C4b upregulation (especially in the brain with high cyst burden), C5aR and C3aR upregulation in the cerebral cortex and glial cells	[53,63,73]
C3, C4, C1q and C1r upregulation in the brain with high cyst burden (complement deposition on the surface of degenerating neurons)	[73]
C1q upregulation (especially near parasite cysts and punctate synaptic patterns)	[53,74]
C3, C4b, and C1q upregulation and the probable induction of the disruption of tight junctions	[53]
Induction or upregulation of the alternative pathway components (FB and FP) and anaphylatoxin receptors (C3aR and C5aR) in the cerebral cortex and glial cells	[63]

### 2.3. Trypanosoma cruzi

*Trypanosoma cruzi* is the etiologic agent of Chagas disease (American trypanosomiasis), and is mainly transmitted to vertebrate hosts by feces or urine of infected blood-sucking triatomine bugs. Blood trypomastigote forms can migrate via the bloodstream and infect organs including the heart, esophagus, and stomach of the vertebrate hosts [75]. Complement cascade may play in humans a dual function during acute and chronic phases of Chagas disease. It might exert important functions in parasite elimination in the initial steps of the infection, and in the later stages it might be involved in the progression of symptomatic forms of the disease through its role in T-cell regulation [76]. *Trypanosoma* parasites mostly trypomastigotes and amastigotes adapt to the immune and complement system through different strategies. Trypomastigotes seem to be remarkably resistant to the complement and this resistance vary between strains of the parasites [77].

The alternative and lectin complement pathways are activated In *T. cruzi* in initial steps of the infection. In general, *T. cruzi* recruits calreticulin and glycoprotein 58/68 (GP58/68) biomolecules to inhibit the initial steps of CP, LP or AP complement pathways, respectively, and trypomastigote-decay acceleration factor (T-DAF), complement C2 receptor inhibitor trispanning (CRIT), complement regulatory protein (CRP), and host-derived microvesicles (MVs) that inhibit or disrupt C3 convertase assembly, leading to the complement evasion of the parasite [77].

Different infective forms of *T. cruzi* can express or mimic several complement regulatory proteins on their surfaces. It has been described that the expression level (mRNA or protein) of some complement receptors or complement-inhibiting proteins, including CRP, CRIT and T-DAF could be alleviated in *T. cruzi* strains (trypomastigotes) with lower virulence. Thus, the differential expression of such complement regulatory proteins could be valuable diagnostic markers in the assessment of parasite-strain pathogenicity [78]. Most of these expressed proteins have a decay-accelerating activity through the inhibition of C3 convertase formation or assembly (including T-DAF, *T. cruzi* complement regulatory protein (TcCRP), gp58/68 and FH) and/or contribute as a cofactor for complement inhibitor factor I (FI). Some of them can also restrict the catalytic activity of C3 convertase (plasma membrane-derived vesicles (PMVs)) or inactivate and inhibit specific complement components (*T. cruzi* calreticulin (TcCRT) and CRIT). TcCRT as a pleiotropic and multifunctional molecule could be expressed in both parasite endoplasmic reticulum and parasite surface, participating in parasite pathogenesis especially in correlation with the complement cascade. This protein can disrupt the initial step of CP and LP pathways, decreasing the C3 convertase formation. TcCRT can also exhibit important virulence functions in correlation with C1 [79]. In addition, it has been observed that some *T. cruzi* strains hijack complement relevant proteins such as opsonized components of the complement, such as mannose-binding lectins (MBLs), leading to host cells invasion [80]. 

Extracellular vesicles (EVs) including MVs or exosomes might be released into different cells by many pathogens, such as intracellular protozoan parasites during parasite–host cell interaction and transfer different biomolecules as virulence factors [81]. MVs cargo can lead to parasite resistance to complement-mediated lysis and drive parasite invasion and infection induction in a parasite class dependent manner [82,83]. Furthermore, these strategies appear to be parasite-specific and also parasite-strain independent [81,83,84]. For instance, calreticulin in MVs binds to subcomponents of the classical and lectin pathways, inhibiting the formation of C3 convertase, restricting the activation of classical and lectin pathways, and eliminating any complement system membrane attack complex located on the parasite´s surface [84,85]. 

### 2.4. Trypanosoma brucei

African trypanosomiasis (or sleeping sickness) is caused by protozoan parasites of the species *Trypanosoma brucei*. This infectious disease is transmitted by the tsetse fly (*Glossina* species). Two subspecies of the parasite (morphologically indistinguishable) *T. b. gambiense* and *T. b. rhodesiense* induce chronic (slowly progressing) African trypanosomiasis in western and central Africa and acute African trypanosomiasis in eastern and southern Africa, respectively [86]. 

In African trypanosomes, both the classical (mediated by specific antibodies against the parasite) and alternative (antibody-independent and in the early stages of infection) pathways of the complement are activated during infection [87]. It seems that the complement system activation is favorable but not essential for resistance to the infection. In fact, C3a and C5a have been recognized as helpful and efficient complement factors in the initiation of inflammatory responses during trypanosomes infection [88]. As a defensive mechanism, trypanosomes can endocytose surface bound anti-variant surface glycoprotein (VSG) specific antibodies (lytic complement complexes) through the flagellar pocket [89]. The metacyclic infectious trypanosomes are able to express an VSG coating antigen that inhibits antibodies-mediated elimination. This mosaic VSG coat also inhibits the recognition of hidden epitopes of complement factors (C3) to their surface. Furthermore, the Ab-mediated elimination could be restricted due to the rapid recycling of VSG-antibody complexes and VSG shedding, driven to scavenging circulating complement components. Therefore, it has been speculated that by their VSG recycling system and by releasing large amounts of soluble VSG, especially at the peak of parasitemia, a hypocomplementemia phenomenon is induced during infection, leading to parasite survival and immune escape of the parasites [87,88,90].

On the other hand, it is very interesting to point out that the host´s lipids scavenging and manipulation by some pathogens allow them to evade the immune system, promoting survival and persistence through different mechanisms [91]. As a postulated mechanism, pathogenic or host’s hijacked phospholipids might interact with various complement components including FH and FHL and manipulate this pivotal cascade to favor inducing the infection [92]. It has been mentioned that *T. brucei* infection triggers a broad and robust immune response in the adipose tissue (AT), which needs the complement cascade (C3 component) to diminish the tissue-parasitic burden [93]. In parasitic infections, the host’s lipid scavenging brings the parasite essential metabolic enzymes. In addition, in some protozoan parasites including *Trypanosoma* and *Plasmodium* spp. It also represents countermeasures for the neutralization of the host’s complement effects [94].

### 2.5. Leishmania spp.

Leishmaniasis is a vector-borne disease caused by 53 species belonging to the genus *Leishmania*. Moreover, 20 species are described as pathogenic to humans. It is estimated that 50.000–90.000 new cases of visceral leishmaniasis and 600.000 up to 1 million cases of cutaneous leishmaniasis occur annually in the world [95]. 

As in other protozoan parasites, several leishmanial proteins, including membrane-associated protein inhibitors expressed on *Leishmania amazonesis* complement-resistant promastigotes (GP63, GP46) and inhibitors of serine proteinase (ISP) are involved in the modulation or inhibition of the host’s complement system by different mechanisms during *Leishmania* infections. Alterations in lipophosphoglycan (LPG) structure also modulate the susceptibility of *Leishmania* metacyclic promastigotes against the complement. It seems that a developmental change in the LPG inhibits the integration of C5b-9 into the metacyclic promastigotes membrane doing them less susceptible to complement lysis [95,96]. Furthermore, recent data suggest that *L. amazonensis* metacyclic promastigotes are able to repair the lytic pores induced by MAC facilitating parasite survival against the lytic effect of the complement [95]. 

In addition, a protein kinase-1 (LPK-1) isolated from *L. major* promastigotes might phosphorylate C3, C5, and C9. Interestingly, phosphorylated C3 had been described to be more resistant to cleavage by trypsin than non-phosphorylated C3 [97]. Therefore, such a phosphorylation probably regulates the susceptibility of complement components to proteolytic cleavage, leading to protease protection and complement activation and parasite lysis. *Leishmania* promastigote surface antigen 2 (PSA-2) in cooperation with proteophosphoglycan (PPG) (sharing leucine repeat motifs with PPG) can attach to the CR3, thus facilitates the parasite invasion into the host cells. Since *Leishmania* LPG, PPG, and PSA-2 biomolecules are expressed both in secretions and as membrane-bound proteins, more investigations might confer a complement evasion function for PSA-2 in *Leishmania* parasites [98,99].

During the infectious process, it has been recently described that the promastigotes of the *L. infantum*, *L. braziliensis*, and *L. amazonensis* hijack the FH (and probably FH-related proteins), as cofactor for FI-mediated C3b cleavage. Although this strategy is similar to that of GP63 (through C3b inactivation), they work in independent ways. The parasitic protein GP46 is considered as a plausible target that interacts with FH. It seems that the enhancement of *Leishmania* survival during infection is related to C3b inhibition or inactivation, the presence of *Phlebotomus* salivary complement inhibitors and the ability to decrease the complement levels by less resistant parasites [100]. 

Table 2 represents different mechanisms of action used by some protozoan parasites (including *Plasmodium*, *Trypanosoma* and *Leishmania* spp.) during their interaction with the host’s complement systems.

### 2.6. Growing Evidences Supporting Complement Modulation by Entamoeba, Giardia and Trichomonas spp.

Several proteins from other pathogenic protozoa such as *Entamoeba histolytica, Giardia lamblia* and *Trichomonas vaginalis* involved in complement modulation and related mechanisms have been identified. *E. histolytica* galactose and N-acetyl-D-galactosamine (Gal/GalNAclectin) protein prevents the formation of MAC in the parasite membrane and secretory cysteine-proteases inhibiting the anaphylatoxins C3a and C5a [95]. In addition, *E. histolytica* exports the host FI cofactor CD46 and the MAC inhibitor CD59 to the outer of their plasma membranes [112]. This parasite also removes the lytic pore (induced by the secretion of acid sphingomyelinases from lysosomes) on the plasma membrane through endocytosis or probably by detaching from the plasma membrane through vesicular budding and consequently evades the host’s complement system [95]. 

During giardiasis, the upregulation of IL-17A is required for the release of IgA into the lumen of the host intestine, for the production of antimicrobial peptides and the regulation of complement activation [113]. Detailed steps of such a process remain unclear although it could be postulated that *Giardia* parasites might use sophisticated strategies to induce the complement evasion. 

Interestingly, after *Trichomonas vaginalis* infection the parasite acquires CD59 from various host cells (such as RBCs) to decrease C9 polymerization and to modulate complement system [114].

## 3. Potential Targets Based on Protozoa-Host’s Complement Interactions to Manage Neglected Diseases 

It is clear that the antibody response together with the complement cascade could be an important contributor to natural acquired immunity against protozoan parasites leading to an effective complement attack [7,16,21]. Relevant vaccine antigens and humoral responses, such as anti-CSP antibodies, including IgG1, IgG3, and IgM, can fix the complement system and activate the CP pathway [115,116,117]. Thus, humoral responses are expected to include complement-fixing antibodies to neutralize the parasites. Therefore, different and multiple targets for complement fixing antibodies on different life cycle stages of protozoan parasites (*P. falciparum* reticulocyte binding protein homologue 5 (PfRH5), PfRH2, glycosylphosphatidylinositol (GPI)-anchored micronemal antigen (GAMA), merozoite surface protein duffy binding ligand 1 (MSP-DBL1), MSP2-3D7, erythrocyte-binding antigen 175 (EBA-175), EBA140 in merozoite and Pfs230 in gametocytes of *Plasmodium*) further increase the potentiality of the vaccine cocktails for the induction of complement-mediated antibody responses able to further reduce the possibility of complement resistance of parasitic antigens [21,118]. The involvement of complement-fixing antibodies in the mechanism action of the most advance malaria vaccine RTS,S and in a whole sporozoite-based vaccine (Sanaria PfSPZ) further highlight the prominent role of the antibody response in a complement-dependent in designing effective vaccines [117,119,120].

The use of subunit vaccines, including invariant flagellum antigen from *T. vivax* (IFX) has shown that the recombinant monoclonal antibodies to this protein (anti-IFX antibodies) induce sterile and long-lasting immunity and inhibit parasite replication through different mechanisms of antibody-mediated immunity, especially the dominant role of complement system [121]. In passive protection experiments the authors demonstrated that a mutation in the host C1q binding site approximately restored the inhibition of parasite growth, suggesting that C1q-mediated complement recruitment is a crucial protective mechanism of this vaccine. Accordingly, it seems that more investigations on the vaccine mechanisms related to the complement system can further increase the efficacy of vaccine strategies against intracellular protozoan parasites, such as trypanosomes. 

As aforementioned, the VSG coat is highly antigenic in African trypanosomes and induces robust anti VSG-specific antibodies, participating in the opsonization and parasite lysis mediated by the complement. It is well known that some trypanosomes (*T. brucei*) are resistant to lysis mediated by the complement, and this event is triggered by coating of VSG on the surface of the parasite [87,88,90]. *T. brucei* major surface proteases (TbMSPs) and other enzymes, such as Phospholipase-C (PLC), are responsible for the cleavage and release of VSG from the parasite surface. Accordingly, the use of TbMSPs and TbPLC inhibitors or TbMSPs and TbPLC-gene knockout methods could be an attractive strategy to inhibit the release of VSG molecules and subsequently to block the mechanism of antigenic variation. These approaches might then drive to the chronicity of infection, a better management of the parasite–host interaction and finally the development of novel therapeutic strategies, inhibiting or selective regulating the release of VSG and manipulating the complement system to decrease or abolish the parasitemia [122,123]. However, the mechanisms of action and regulation of TbMSPs of *T. brucei* are still elusive and will need more experiments. In addition, vaccination with important antigens for the modulation and neutralization of the host complement system, such as MSP and PLC, might generate monoclonal antibodies that interfere or block the VSG shedding mechanisms in African trypanosomes [123].

Regarding therapeutic interventions, the recruitment of complement regulatory protein FH is probably the most complement-inhibiting mechanism in different pathogens, especially intracellular pathogens, including protozoan parasites [7,8,124]. Thus, several strategies, including (i) interference with pathogen sialic acid expression (using sialic acid analogs unable to recognize FH), (ii) usage of competitive inhibitors to functionally disable factor H binding proteins (FHBP), (iii) identification of factor H-Fc fusion proteins leading to receptor-mediated phagocytosis, and (iv) use of inhibitors to restrict conformational change of FHBP essential to augment their binding to FH could be considered therapeutic insights in clinical approaches [8]. 

Recent data propose that the natural change in FH plasma levels correlated with malaria severity and susceptibility. Thus, the interference on the binding of FH to *P. falciparum* parasite might be utilized for malaria prevention or treatment [125]. Additionally, the protozoan parasites (such as *T. brucei*) express distinct receptors on their surface that might exploit the mammalian FH to enhance parasite transmission to their insect vectors. This phenomenon allows the inhibitory domains of FH to remain free and to further inhibit complement C3b deposited on the parasite surface [126]. Accordingly, the design of some inhibitors or specific antibodies against such proteins (FHB parasite receptors) might suggest novel therapeutic strategies against protozoan parasitic transmission. 

Several other parasitic proteins including Pfs47, GAP50, and PIMMS43 can facilitate *Plasmodium* parasites transmission in mosquitoes by its capability to modulate the insect complement-like immune system. Thus, the identification of such proteins and their receptors on midgut cells from different protozoan parasites vectors might facilitate biological therapeutic insights and transmission-blocking vaccines. In this sense, some vaccine candidates including Pfs25 and Pfs25-IMX313/Matrix-M (IMX313 as a hybrid of the oligomerization domain of chicken complement inhibitor C4-binding protein -C4bp-) have been described as plausible malaria transmission-blocking candidate vaccines [127]. 

When dealing with American trypanosomiasis, *T. cruzi* trypomastigotes are normally resistant to complement-mediated lysis once treated with trypsin and sialidase. Interestingly, their susceptibility to the complement-mediated death increased in human serum. These data indicate that parasites hijack FH through surface-bound sialic acid with an unknown mechanism [128]. 

On the other hand, *T. gondii* probably recruits FH through the expression of heparan sulfated proteoglycans or sialic acid [50]. Some vaccines had been approved based on the FHBPs against group B meningococcus [129]. Besides activating the complement system, FHBP-specific antibodies can block the binding site for FH, leading to an increased pathogen susceptibility to removal through the alternative pathway [7,29]. This vaccine strategy has been appropriately described and developed in bacterial infections compared to other infections. However, some of the parasitic proteins expressed on different developmental life cycle-stages of parasites (including MSP3.1, GPI and Pf92 in *P. falciparum* (as FHBPs)) can also exert pivotal functions in binding or attachment of the parasite to the complement regulatory proteins such as FH and FHL-1 [7]. 

It is known that *T. gondii* disrupts the TJ of BBB and facilitates parasite to pass into the CNS mediated by the crucial role of complement components such as C3 [53,54]. Since the overexpression or overactivation of different complement components (or parasite-host’s complement interaction) has been reported in *Toxoplasma* (Table 1) [51,53,63], the pharmacological or genetic suppression of such critical complement components may decrease the damage in the host and suggesting a therapeutic opportunity. However, the complement cascade probably induces other different balances in the brain physiology (crucial homeostatic roles in the CNS, such as the elimination of apoptotic cells, and toxic substances, cell debris, etc.) and also plays a double-edged sword in the toxoplasmosis pathogenicity and immunity [12,130]. Therefore, more studies are required to identify specific inhibitors based on this hypothesis. Since an association was found between C3 concentration and the number of abortion during *Toxoplasma* infection in pregnant women [131], this information might also be insightful for designing therapeutic targets or diagnostic tools in congenital toxoplasmosis. In addition, *Toxoplasma* infection can increase neural cell death, alteration in neural gene expression and the release of inflammatory mediators in neurospheres [132]. Thus, this infection could be correlated with psychiatric diseases and human CNS disorders including Alzheimer, dementia, depression and schizophrenia [54,133,134,135,136]. On the other hand, due to the overexpression of complement components in *Toxoplasma*-infected brain [53,63], and the upregulation of C1q, C3, and C4b in some mental disorders including schizophrenia, alzheimer’s disease, aging and multiple sclerosis [54,137,138,139,140], it could be interesting to focus on therapeutic studies that share more information regarding the possible correlations between parasitic disease including toxoplasmosis and mental disorders and their association with the complement system.

Other several parasitic proteins or antigens have been also described as proteins that might regulate the host’s complement system, suggesting as plausible therapeutic targets. The levels of CK1.2, expressed in *Leishmania* parasites, could be correlated with the infection and host cell immune response subversion. *Leishmania* CK1.2 can directly phosphorylate human complement component C3a or the human interferon alpha and beta receptor subunit 1 (IFNAR1) and consequently suppresses immune responses. In this sense, the use of CK1 inhibitors remarkably decreased or blocked the growth of *Leishmania* parasites [97,111,141]. SPECT1 and SPECT2 (sporozoite microneme protein essential for cell traversal) have been described as *Plasmodium* sporozoites proteins essential for cell traversal during the infection. SPECT2 carries a MAC/perforin-related domain to induce pores -mediated by complement components- in the infected host cell membrane probably allowing the sporozoites to cross the hepatocytes [142,143,144]. Therefore, the design of inhibitors against these crucial parasitic proteins, might be therapeutically useful. Moreover, *P. falciparum* LCCL domain-containing protein 1 (PfCCp1) (belonging to a multi-domain protein family called the LCCL domain-containing proteins -CCp- or LCCL/lectin adhesive-like proteins -LAPs-) inhibits the activation of the classical complement pathway and down-regulates effector responses of dendritic cells, highlighting a major role for PfCCp1 and related proteins in modulating the host’s immune or complement system and parasite evasion [145,146]. Although this parasitic protein seems to be a putative therapeutic target, another study showed that CCp/LAP knockout parasites progressed similar to wild-type parasites in asexual and sexual stage development, probably indicating the primary role of these proteins is unlikely to be related to the parasite evasion from the complement system or the presence of redundant mechanisms [146]. More investigations are required to corroborate the therapeutic function of these proteins in malaria infection. 

## 4. Concluding Remarks and Future Perspectives

The complement system can induce both beneficial and deleterious functions in parasitic infections. For instance, the complement system plays both valuable and harmful functions during *Leishmania* infections. In fact, the complement mediates lysis by MAC (parasite elimination), whereas opsonization via C3b/iC3b increases phagocytic activity (inducing parasite internalization and survival) [100]. Different pathogens including protozoa may develop strategies to evade complement and to neutralize the complement lysis or killing effects. Currently, complement evasion has been highlighted as an essential strategy in the parasite–host interaction and during the progression of protozoan parasitic infections. It seems that the mimicking or hijacking of the complement components is a prominent and common phenomenon used by the abovementioned parasites. 

Since protozoan parasites have several strains and multiple developmental life cycle stages; their mechanisms of action against the complement system are also different and most probably redundant. For instance, different strains of *Trypanosoma* parasites can express different complement receptors or complement-inhibiting proteins (such as CRP, T-DAF, and CRIT) and restrict different pathways (e.g., classic, alternative and lectin) of the complement [78]. Moreover, the expression of various surface proteins in different *Toxoplasma* strains may affect the activation of the complement system and C3b deposition [50]. Furthermore, hosts (humans versus animals) and the microenvironmental conditions seem to influence parasite–complement cascade interactions (parasite complement evasion or susceptibility or resistance to complement). Therefore, such mechanisms of complement resistance or evasion of several protozoa are different compared with those described in their insect vectors. 

The identification of the source of parasitic antigens that interact with and modulate the host’s complement system should also be addressed and investigated for a better understanding of the biological aspects of parasite–host complement interactions, designing more effective vaccines, and identifying drug targets. Interestingly, EVs (MVs or exosomes) release by different pathogens including intracellular protozoa and subsequently transfer multiple virulence factors and biomolecules to host cells, driven to the alteration of host’s susceptibility to infection [81]. Different protozoan parasites (and their relevant strains) might exhibit diverse grades of resistance and sensitivity to complement-mediated lysis through shedding different levels of MVs, thereby produce heterogeneous phenotypic effects during infections [84]. Some biomolecules, including GP82, GP85 and GP63, isolated from EVs of trypanosomatids, exert crucial functions in the communications between parasites and the host complement system. For example, GP82 probably modulates the host immune system and facilitates cell attachment and regulates the host complement system in *T. cruzi* infection. It was also described that GP85 can modulate transforming growth factor beta (TGF-β)-bearing EVs released from host cells, leading to *T. cruzi* escaping the complement attack. Moreover, GP63 induces *Leishmania* parasites (*L. donovani*) evasion by modulating complement mediated lysis and enhancing parasite phagocytosis [82,103]. Several proteins, including TS/gp85 superfamily members (gp85/Trans-Sialidase Superfamily of Glycoproteins), α-galactosyl-containing glycoconjugates, proteases, MASPs, and cytoskeleton proteins, have also been detected as the main components of the *T. cruzi*-derived vesicles [147]. More data are needed to corroborate their interaction with the host’s complement system. Thus, due to the important role of EVs and MVs in the immune evasion of parasites (such as trypanosomatids) [103] and also the abovementioned information, the identification of such biomolecules that have originated from EVs and MVs can further suggest the potential role of such excretory-secretory fractions of parasites in the recognition of parasitic proteins that regulate the host’s complement during parasitic infections. 

Considering the expression of different polymorphisms in complement components, such as complement regulator proteins (FH and CR1 polymorphisms) [11,148,149], the potential implications of these variations on complement evasion mechanisms of the parasites should be also analyzed in future studies and further developed as an interesting challenge during parasitic infections. In contrast to this event, different pathogens including protozoan parasite might also exhibit different motifs in their pathogenic-antigens to update their interactions with complement components (to improve the evasion mechanism) [150]. More investigations and data are required to confirm these insights during protozoan parasitic infections.

The use of modern proteomic-based approaches might shed light on the pathogen or parasite-host’s complement interactions by recognizing novel essential molecular interactions, serving as targets to deal with infections [151]. The analysis of the proteome of MVs released by *T. cruzi* trypomastigotes indicated that *T. cruzi*-MVs contain complement resistant proteins, such as calreticulin [152]. Interestingly, the use of proteomics has also elucidated the upregulation of several complement components factors, such as C1q and C3 in *Toxoplasma*-infected brain tissues and their correlations with parasite pathogenicity [53]. Interestingly, using immunoproteomic techniques, C1QBP (complement component 1 Q subcomponent-binding protein) has been identified in *L. infantum* amastigotes extracted from infected macrophages [153]. Very recent information has shown that *Toxoplasma*-rhoptry protein 1 (ROP1) interacts with the host cell protein C1QBP and probably induces parasite immune evasion. ROP1 can alleviate C1QBP and the absence of ROP1 enhances the susceptibility to interferon gamma (IFNγ)-mediated restriction. Therefore, ROP1 and C1QBP are crucial for higher resistance to IFNγ-mediated restriction [154]. Moreover, since different lipoproteins increase pathogen survival by binding or inactivating complement pathways [92]; the use of different proteomic techniques can reveal more information regarding the structure and function of pathogenic antigens and complement components and consequently the pathogen–host’s complement interaction [155].

The intracellular activation of complement system (complosome) may also affect crucial intracellular signaling pathways, facilitating pathogen deletion [95]. These important intracellular functions of complement cascade have not been appropriately described in pathogens, including protozoan parasites, therefore, further studies need to be performed.

## Figures and Tables

**Table 2:** Interaction of protozoan parasites with the host’s complement systems through different mechanisms.

Biomolecules, Proteins, Receptors	Mechanism of Action to Inhibit Complement	References
*Plasmodium* spp.		
MSP3.1	The inactivation of C1s and mucin-associated surface protein-2 (MASP-2) through C1-INH	[32]
Mannosyltransferase (PIG-M)	Employing CD59 to decrease C9 polymerization on the cell surface by binding to C8a and C9	[101]
*T. cruzi*		
Calreticulin (TcCRT)	Interacts with L-ficolin and preventing C4 conversion to C4b, interacting with C1q collagen-like domain (inhibiting both the classical and lectin pathways)	[5,14,77,79,95,102,103,104,105]
*T. cruzi* complement regulatory protein (TcCRP)	Binding to C3b and C4b and inhibiting the formation of the classical and alternative complement C3 convertase	
*T. cruzi* complement C2 receptor inhibition trispanning (TcCRIT)	Blocking C2 cleavage by C1s or MASP-2 into C2a and inhibiting C3 convertase formation, hijacking C2 (modulating the activation of the lectin and classical complement pathways)	
Trypomastigote decay-accelerating factor (T-DAF)	Mimicking the activity of the complement regulatory protein DAF, blocking C3 and C4, accelerating the dissociation or assembly of C3 convertases (modulating/inhibiting the activation of the alternative, classical, and probably the lectins pathways)	
Glycoprotein 58/68	Preventing the formation of cell-bound C3 convertase (decay-accelerating activity) by inhibiting the initial association of factor B (FB) to surface fixed C3b, attaching to human complements C3b and C4b to prevent the activation of the complement)	
Membrane-derived vesicles (microvesicles)	Inhibiting the classical and lectin pathways by binding to C3 convertase C4b2a on the parasite surface and decreasing its catalytic activity	
N- and O-glycosylated biomolecules	To inhibit activation of the lectin complement pathway through L-ficolin, H-ficolins, and mannose-binding lectin (MBL) (resulting in the failure of MASP-2-induced C2 and C4 cleavage)	
GP72	Inhibiting the formation of the C3 convertase in the alternative pathway	
GP160	As a member of the C3/C4 binding family of complement regulators: Inhibits the formation of the alternative and classical C3 convertase (preventing the activation of the complement cascade)	
*Leishmania* spp.		
GP63	Cleaving parasite-bound C3b into inactive form iC3b, (prevent the formation of C3 and C5 convertase and the MAC-mediated lysis of the parasite)	[5,95,106,107]
GP46 (as a membrane-associated protein inhibitors expressed on *L. amazonensis* complement-resistant promastigotes)	Inhibiting the lytic activity of AP, impairing C9, but not C3, attaching to complement-activating complex (probably block the complement activation after C3b deposition and at the stage of C9 deposition)	[95,108]
Inhibitors of serine proteinase (ISP) (*L. donovani*)	Interacting with host C1r, C1s, MASP-1 and MASP-2, preventing the formation of CP and LP initiators (preventing the formation of C3 convertase, decreasing the production of the anaphylatoxins C3a and C5a)	[5,95,109,110]
Casein kinase 1 isoform 2 (CK1.2)	Interacting with C3a and modulating complement system and inducing immune evasion	[97,111]

## References

[1] Kellermann M., Scharte F., Hensel M. (2021). Manipulation of Host Cell Organelles by Intracellular Pathogens. Int. J. Mol. Sci..

[2] Sakamoto H., Nakada-Tsukui K., Besteiro S. (2021). The Autophagy Machinery in Human-Parasitic Protists; Diverse Functions for Universally Conserved Proteins. Cells.

[3] Bosurgi L., Rothlin C.V. (2021). Management of cell death in parasitic infections. Semin. Immunopathol..

[4] Ivanova D.L., Denton S.L., Fettel K.D., Sondgeroth K.S., Munoz Gutierrez J., Bangoura B., Dunay I.R., Gigley J.P. (2019). Innate lymphoid cells in protection, pathology, and adaptive immunity during apicomplexan infection. Front. Immunol..

[5] Chulanetra M., Chaicumpa W. (2021). Revisiting the Mechanisms of Immune Evasion Employed by Human Parasites. Front. Cell. Infect. Microbiol..

[6] Merle N.S., Church S.E., Fremeaux-Bacchi V., Roumenina L.T. (2015). Complement system part I–molecular mechanisms of activation and regulation. Front. Immunol..

[7] Kiyuka P.K., Meri S., Khattab A. (2020). Complement in malaria: Immune evasion strategies and role in protective immunity. FEBS Lett..

[8] Moore S.R., Menon S.S., Cortes C., Ferreira V.P. (2021). Hijacking factor H for complement immune evasion. Front. Immunol..

[9] Jagatia H., Tsolaki A.G. (2021). The Role of Complement System and the Immune Response to Tuberculosis Infection. Medicina.

[10] Bardhan M., Kaushik R. (2019). Physiology, Complement Cascade. StatPearls.

[11] Schmidt C.Q., Kennedy A.T., Tham W.-H. (2015). More than just immune evasion: Hijacking complement by *Plasmodium falciparum*. Mol. Immunol..

[12] Sikorski P.M., Commodaro A.G., Grigg M.E. (2021). A protective and pathogenic role for complement during acute *Toxoplasma gondii* infection. Front. Cell. Infect. Microbiol..

[13] Leitner W.W., Haraway M., Pierson T., Bergmann-Leitner E.S. (2020). Role of opsonophagocytosis in immune protection against malaria. Vaccines.

[14] Shao S., Sun X., Chen Y., Zhan B., Zhu X. (2019). Complement evasion: An effective strategy that parasites utilize to survive in the host. Front. Microbiol..

[15] World Health Organization (2021). Ending the neglect to attain the Sustainable Development Goals: A road map for neglected tropical diseases. Front. Cell. Infect. Microbiol..

[16] Rathnayake D., Aitken E.H., Rogerson S.J. (2021). Beyond Binding: The Outcomes of Antibody-Dependent Complement Activation in Human Malaria. Front. Immunol..

[17] Elmahallawy E.K., Alkhaldi A.A., Saleh A.A. (2021). Host immune response against leishmaniasis and parasite persistence strategies: A review and assessment of recent research. Biomed. Pharmacother..

[18] Yasmin H., Adhikary A., Al-Ahdal M.N., Roy S., Kishore U. (2022). Host–Pathogen Interaction in Leishmaniasis: Immune Response and Vaccination Strategies. Immuno.

[19] Okunlola O.A., Oyeyemi O.T. (2022). Malaria transmission in Africa: Its relationship with yellow fever and measles. PLoS ONE.

[20] Al-Awadhi M., Ahmad S., Iqbal J. (2021). Current status and the epidemiology of malaria in the Middle East Region and beyond. Microorganisms.

[21] Kurtovic L., Boyle M.J., Opi D.H., Kennedy A.T., Tham W.H., Reiling L., Chan J.A., Beeson J.G. (2020). Complement in malaria immunity and vaccines. Immunol. Rev..

[22] Dinko B., Pradel G. (2016). Immune evasion by *Plasmodium falciparum* parasites: Converting a host protection mechanism for the parasite′ s benefit. Adv. Infect. Dis..

[23] Boyle M.J., Reiling L., Feng G., Langer C., Osier F.H., Aspeling-Jones H., Cheng Y.S., Stubbs J., Tetteh K.K., Conway D.J. (2015). Human antibodies fix complement to inhibit *Plasmodium falciparum* invasion of erythrocytes and are associated with protection against malaria. Immunity.

[24] Boyle M., Reiling L., Beeson J. (2016). Evaluating Complement-Mediated Humoral Immunity to *P. falciparum* Blood Stages. EBioMedicine.

[25] Biryukov S., Angov E., Landmesser M.E., Spring M.D., Ockenhouse C.F., Stoute J.A. (2016). Complement and antibody-mediated enhancement of red blood cell invasion and growth of malaria parasites. EBioMedicine.

[26] Akhouri R.R., Goel S., Furusho H., Skoglund U., Wahlgren M. (2016). Architecture of human IgM in complex with *P. falciparum* erythrocyte membrane protein 1. Cell Rep..

[27] Kennedy A.T., Schmidt C.Q., Thompson J.K., Weiss G.E., Taechalertpaisarn T., Gilson P.R., Barlow P.N., Crabb B.S., Cowman A.F., Tham W.-H. (2016). Recruitment of factor H as a novel complement evasion strategy for blood-stage *Plasmodium falciparum* infection. J. Immunol..

[28] Simon N., Friedrich O., Kappes B. (2018). Quantification of human complement factor H binding to asexual malaria blood stages by an enzyme-linked immunosorbent assay. Vaccine.

[29] Rosa T.F., Flammersfeld A., Ngwa C.J., Kiesow M., Fischer R., Zipfel P.F., Skerka C., Pradel G. (2016). The *Plasmodium falciparum* blood stages acquire factor H family proteins to evade destruction by human complement. Cell. Microbiol..

[30] Cserhalmi M., Papp A., Brandus B., Uzonyi B., Józsi M. (2019). Regulation of regulators: Role of the complement factor H-related proteins. Semin. Immunol..

[31] Reiss T., Thiago F.d.A., Blaesius K., Bobbert R.P., Zipfel P.F., Skerka C., Pradel G. (2018). Cutting edge: FHR-1 binding impairs factor H–mediated complement evasion by the malaria parasite *Plasmodium falciparum*. J. Immunol..

[32] Kennedy A.T., Wijeyewickrema L.C., Huglo A., Lin C., Pike R., Cowman A.F., Tham W.-H. (2017). Recruitment of human C1 esterase inhibitor controls complement activation on blood stage *Plasmodium falciparum* merozoites. J. Immunol..

[33] Mejia P., Diez-Silva M., Kamena F., Lu F., Fernandes S.M., Seeberger P.H., Davis III A.E., Mitchell J.R. (2016). Human C1-inhibitor suppresses malaria parasite invasion and cytoadhesion via binding to parasite glycosylphosphatidylinositol and host cell receptors. J. Infect. Dis..

[34] Oyong D.A., Kenangalem E., Poespoprodjo J.R., Beeson J.G., Anstey N.M., Price R.N., Boyle M.J. (2018). Loss of complement regulatory proteins on uninfected erythrocytes in vivax and falciparum malaria anemia. JCI Insight.

[35] Horta M.F., Andrade L.O., Martins-Duarte É.S., Castro-Gomes T. (2020). Cell invasion by intracellular parasites–the many roads to infection. J. Cell Sci..

[36] Touray M.G., Seeley D., Miller L.H. (1994). *Plasmodium gallinaceum*: Differential lysis of two developmental stages of malaria sporozoites by the alternative pathway of complement. Exp. Parasitol..

[37] Wiesner J., Jomaa H., Wilhelm M., Tony H.P., Kremsner P.G., Horrocks P., Lanzer M. (1997). Host cell factor CD59 restricts complement lysis of *Plasmodium falciparum*-infected erythrocytes. Eur. J. Immunol..

[38] Larsen M.D., Quintana M.d.P., Ditlev S.B., Bayarri-Olmos R., Ofori M.F., Hviid L., Garred P. (2019). Evasion of classical complement pathway activation on *Plasmodium falciparum*-infected erythrocytes opsonized by PfEMP1-specific IgG. Front. Immunol..

[39] Reiss T., Theis H.I., Gonzalez-Delgado A., Vega-Rodriguez J., Zipfel P.F., Skerka C., Pradel G. (2021). Acquisition of human plasminogen facilitates complement evasion by the malaria parasite *Plasmodium falciparum*. Eur. J. Immunol..

[40] Ayón-Núñez D.A., Fragoso G., Bobes R.J., Laclette J.P. (2018). Plasminogen-binding proteins as an evasion mechanism of the host’s innate immunity in infectious diseases. Biosci. Rep..

[41] Simon N., Lasonder E., Scheuermayer M., Kuehn A., Tews S., Fischer R., Zipfel P.F., Skerka C., Pradel G. (2013). Malaria parasites co-opt human factor H to prevent complement-mediated lysis in the mosquito midgut. Cell Host Microbe.

[42] Sologub L., Kuehn A., Kern S., Przyborski J., Schillig R., Pradel G. (2011). Malaria proteases mediate inside-out egress of gametocytes from red blood cells following parasite transmission to the mosquito. Cell. Microbiol..

[43] Khattab A., Barroso M., Miettinen T., Meri S. (2015). Anopheles midgut epithelium evades human complement activity by capturing factor H from the blood meal. PLoS Negl. Trop. Dis..

[44] Molina-Cruz A., Garver L.S., Alabaster A., Bangiolo L., Haile A., Winikor J., Ortega C., van Schaijk B.C., Sauerwein R.W., Taylor-Salmon E. (2013). The human malaria parasite Pfs47 gene mediates evasion of the mosquito immune system. Science.

[45] Ramphul U.N., Garver L.S., Molina-Cruz A., Canepa G.E., Barillas-Mury C. (2015). *Plasmodium falciparum* evades mosquito immunity by disrupting JNK-mediated apoptosis of invaded midgut cells. Proc. Natl. Acad. Sci. USA.

[46] Molina-Cruz A., Canepa G.E., Kamath N., Pavlovic N.V., Mu J., Ramphul U.N., Ramirez J.L., Barillas-Mury C. (2015). *Plasmodium* evasion of mosquito immunity and global malaria transmission: The lock-and-key theory. Proc. Natl. Acad. Sci. USA.

[47] Zhu F., Zheng H., Chen S., Zhang K., Qin X., Zhang J., Fan Y., Wang L., Li X., Zhang J. (2022). Malaria oocysts require circumsporozoite protein to evade mosquito immunity. Nat. Commun..

[48] Ukegbu C.V., Giorgalli M., Tapanelli S., Rona L.D., Jaye A., Wyer C., Angrisano F., Blagborough A.M., Christophides G.K., Vlachou D. (2020). PIMMS43 is required for malaria parasite immune evasion and sporogonic development in the mosquito vector. Proc. Natl. Acad. Sci. USA.

[49] De Barros R.A.M., Torrecilhas A.C., Marciano M.A.M., Mazuz M.L., Pereira-Chioccola V.L., Fux B. (2022). Toxoplasmosis in Human and Animals Around the World. Diagnosis and Perspectives in the One Health Approach. Acta Trop..

[50] Sikorski P.M., Commodaro A.G., Grigg M.E. (2020). *Toxoplasma gondii* recruits factor H and C4b-binding protein to mediate resistance to serum killing and promote parasite persistence in vivo. Front. Immunol..

[51] Shinjyo N., Kagaya W., Pekna M. (2021). Interaction Between the Complement System and Infectious Agents—A Potential Mechanistic Link to Neurodegeneration and Dementia. Front. Cell. Neurosci..

[52] Olivera G.C., Ross E.C., Peuckert C., Barragan A. (2021). Blood-brain barrier-restricted translocation of *Toxoplasma gondii* from cortical capillaries. Elife.

[53] Huang W.Y., Wang Y.P., Mahmmod Y.S., Wang J.J., Liu T.H., Zheng Y.X., Zhou X., Zhang X.X., Yuan Z.G. (2019). A double-edged sword: Complement component 3 in *Toxoplasma gondii* infection. Proteomics.

[54] Alsaadawi M.A., Alkhuzaie S.S., Alasadiy Y.D., Alsalih N.J., Al-Yasari A.M.R. (2021). Supervision of The Complement System by *Toxoplasma* During Neural Infections (Areview). IOP Conf. Series Earth Environ. Sci..

[55] Jin Y., Yao Y., El-Ashram S., Tian J., Shen J., Ji Y. (2019). The neurotropic parasite *Toxoplasma gondii* induces astrocyte polarization through NFκB pathway. Front. Med..

[56] Nasuhidehnavi A., Yap G.S. (2021). Microglia and astrocyte responses to neuropathogenic protozoan parasites. Fac. Rev..

[57] Carrillo G.L., Ballard V.A., Glausen T., Boone Z., Teamer J., Hinkson C.L., Wohlfert E.A., Blader I.J., Fox M.A. (2020). *Toxoplasma* infection induces microglia-neuron contact and the loss of perisomatic inhibitory synapses. Glia.

[58] Jha M.K., Jo M., Kim J.-H., Suk K. (2019). Microglia-astrocyte crosstalk: An intimate molecular conversation. Neuroscientist.

[59] Cragnolini A.B., Lampitella G., Virtuoso A., Viscovo I., Panetsos F., Papa M., Cirillo G. (2020). Regional brain susceptibility to neurodegeneration: What is the role of glial cells?. Neural. Regen. Res..

[60] Gray S.C., Kinghorn K.J., Woodling N.S. (2020). Shifting equilibriums in Alzheimer’s disease: The complex roles of microglia in neuroinflammation, neuronal survival and neurogenesis. Neural. Regen. Res..

[61] Li S.-M., Li B., Zhang L., Zhang G.-F., Sun J., Ji M.-H., Yang J.-J. (2020). A complement-microglial axis driving inhibitory synapse related protein loss might contribute to systemic inflammation-induced cognitive impairment. Int. Immunopharmacol..

[62] Wang C., Yue H., Hu Z., Shen Y., Ma J., Li J., Wang X.-D., Wang L., Sun B., Shi P. (2020). Microglia mediate forgetting via complement-dependent synaptic elimination. Science.

[63] Shinjyo N., Hikosaka K., Kido Y., Yoshida H., Norose K. (2021). *Toxoplasma* infection induces sustained up-regulation of complement factor B and C5a receptor in the mouse brain via microglial activation: Implication for the alternative complement pathway activation and anaphylatoxin signaling in cerebral toxoplasmosis. Front. Immunol..

[64] Briukhovetska D., Ohm B., Mey F.T., Aliberti J., Kleingarn M., Huber-Lang M., Karsten C.M., Köhl J. (2020). C5aR1 Activation Drives Early IFN-γ Production to Control Experimental *Toxoplasma gondii* Infection. Front. Immunol..

[65] Kumar M., Varun C.N., Dey G., Ravikumar R., Mahadevan A., Shankar S.K., Prasad T.K. (2018). Identification of host-response in cerebral malaria patients using quantitative proteomic analysis. Proteom. Clin. Appl..

[66] Lackner P., Hametner C., Beer R., Burger C., Broessner G., Helbok R., Speth C., Schmutzhard E. (2008). Complement factors C1q, C3 and C5 in brain and serum of mice with cerebral malaria. Malar. J..

[67] Kim H., Erdman L.K., Lu Z., Serghides L., Zhong K., Dhabangi A., Musoke C., Gerard C., Cserti-Gazdewich C., Liles W.C. (2014). Functional roles for C5a and C5aR but not C5L2 in the pathogenesis of human and experimental cerebral malaria. Infect. Immun..

[68] McDonald C.R., Cahill L.S., Ho K.T., Yang J., Kim H., Silver K.L., Ward P.A., Mount H.T., Liles W.C., Sled J.G. (2015). Experimental malaria in pregnancy induces neurocognitive injury in uninfected offspring via a C5a-C5a receptor dependent pathway. PLoS Pathog..

[69] Patel S.N., Berghout J., Lovegrove F.E., Ayi K., Conroy A., Serghides L., Min-Oo G., Gowda D.C., Sarma J.V., Rittirsch D. (2008). C5 deficiency and C5a or C5aR blockade protects against cerebral malaria. J. Exp. Med..

[70] Ramos T.N., Darley M.M., Hu X., Billker O., Rayner J.C., Ahras M., Wohler J.E., Barnum S.R. (2011). Cutting edge: The membrane attack complex of complement is required for the development of murine experimental cerebral malaria. J. Immunol..

[71] Buckingham S.C., Ramos T.N., Barnum S.R. (2014). Complement C5-deficient mice are protected from seizures in experimental cerebral malaria. Epilepsia.

[72] Finley R., Mackey L., Lambert P. (1982). Virulent P. berghei malaria: Prolonged survival and decreased cerebral pathology in cell-dependent nude mice. J. Immunol..

[73] Li Y., Severance E.G., Viscidi R.P., Yolken R.H., Xiao J. (2019). Persistent *Toxoplasma* infection of the brain induced neurodegeneration associated with activation of complement and microglia. Infect. Immun..

[74] Xiao J., Li Y., Gressitt K.L., He H., Kannan G., Schultz T.L., Svezhova N., Carruthers V.B., Pletnikov M.V., Yolken R.H. (2016). Cerebral complement C1q activation in chronic *Toxoplasma* infection. Brain Behav. Immun..

[75] Velásquez-Ortiz N., Herrera G., Hernández C., Muñoz M., Ramírez J.D. (2022). Discrete typing units of *Trypanosoma cruzi*: Geographical and biological distribution in the Americas. Sci. Data.

[76] Caputo M.B., Elias J., Cesar G., Alvarez M.G., Laucella S.A., Albareda M.C. (2022). Role of the Complement System in the Modulation of T-Cell Responses in Chronic Chagas Disease. Front. Cell. Infect. Microbiol..

[77] Cardoso M.S., Reis-Cunha J.L., Bartholomeu D.C. (2016). Evasion of the immune response by *Trypanosoma cruzi* during acute infection. Front. Immunol..

[78] Arroyo-Olarte R.D., Martínez I., Cruz-Rivera M., Mendlovic F., Espinoza B. (2018). Complement system contributes to modulate the infectivity of susceptible TcI strains of *Trypanosoma cruzi*. Mem. Inst. Oswaldo. Cruz..

[79] Ramírez-Toloza G., Ferreira A. (2017). *Trypanosoma cruzi* evades the complement system as an efficient strategy to survive in the mammalian host: The specific roles of host/parasite molecules and *Trypanosoma cruzi* calreticulin. Front. Microbiol..

[80] Evans-Osses I., Mojoli A., Beltrame M.H., Da Costa D.E., DaRocha W.D., Velavan T.P., de Messias-Reason I., Ramirez M.I. (2014). Differential ability to resist to complement lysis and invade host cells mediated by MBL in R4 and 860 strains of *Trypanosoma cruzi*. FEBS Lett..

[81] Gavinho B., Rossi I.V., Evans-Osses I., Inal J., Ramirez M.I. (2018). A new landscape of host–protozoa interactions involving the extracellular vesicles world. Parasitology.

[82] Torrecilhas A.C., Soares R.P., Schenkman S., Fernández-Prada C., Olivier M. (2020). Extracellular vesicles in trypanosomatids: Host cell communication. Front. Cell. Infect. Microbiol..

[83] Deolindo P., Evans-Osses I., Ramirez M.I. (2013). Microvesicles and exosomes as vehicles between protozoan and host cell communication. Biochem. Soc. Trans..

[84] Wyllie M., Ramirez M. (2017). Microvesicles released during the interaction between *Trypanosoma cruzi* TcI and TcII strains and host blood cells inhibit complement system and increase the infectivity of metacyclic forms of host cells in a strain-independent process. Pathog. Dis..

[85] Ferreira V., Valck C., Sánchez G., Gingras A., Tzima S., Molina M.C., Sim R., Schwaeble W., Ferreira A. (2004). The classical activation pathway of the human complement system is specifically inhibited by calreticulin from *Trypanosoma cruzi*. J. Immunol..

[86] Franco J.R., Cecchi G., Paone M., Diarra A., Grout L., Kadima Ebeja A., Simarro P.P., Zhao W., Argaw D. (2022). The elimination of human African trypanosomiasis: Achievements in relation to WHO road map targets for 2020. PLoS Negl. Trop. Dis..

[87] Márquez-Contreras M.E. (2018). Mechanisms of immune evasion by *Trypanosoma brucei*. Microbiol. Curr. Res..

[88] Onyilagha C., Uzonna J.E. (2019). Host immune responses and immune evasion strategies in African trypanosomiasis. Front. Immunol..

[89] Russo D., Williams D., Grab D. (1994). Mechanisms for the elimination of potentially lytic complement-fixing variable surface glycoprotein antibody-complexes in *Trypanosoma brucei*. Parasitol. Res..

[90] Stijlemans B., Caljon G., Van Den Abbeele J., Van Ginderachter J.A., Magez S., De Trez C. (2016). Immune evasion strategies of *Trypanosoma brucei* within the mammalian host: Progression to pathogenicity. Front. Immunol..

[91] Samanta D., Mulye M., Clemente T.M., Justis A.V., Gilk S.D. (2017). Manipulation of host cholesterol by obligate intracellular bacteria. Front. Cell. Infect. Microbiol..

[92] O’Neal A.J., Butler L.R., Rolandelli A., Gilk S.D., Pedra J.H. (2020). Lipid hijacking: A unifying theme in vector-borne diseases. Elife.

[93] Machado H., Bizarra-Rebelo T., Costa-Sequeira M., Trindade S., Carvalho T., Rijo-Ferreira F., Rentroia-Pacheco B., Serre K., Figueiredo L.M. (2021). *Trypanosoma brucei* triggers a broad immune response in the adipose tissue. PLoS Pathog..

[94] Mans B.J., Ribeiro J.M. (2008). A novel clade of cysteinyl leukotriene scavengers in soft ticks. Insect. Biochem. Mol. Biol..

[95] Rios-Barros L.V., Silva-Moreira A.L., Horta M.F., Gontijo N.F., Castro-Gomes T. (2022). How to get away with murder: The multiple strategies employed by pathogenic protozoa to avoid complement killing. Mol. Immunol..

[96] Gupta G., Oghumu S., Satoskar A.R. (2013). Mechanisms of immune evasion in leishmaniasis. Adv. Appl. Microbiol..

[97] Hermoso T., Fishelson Z., Becker S., Hirschberg K., Jaffe C. (1991). Leishmanial protein kinases phosphorylate components of the complement system. EMBO J..

[98] Ouaissi A., Ouaissi M. (2005). Molecular basis of *Trypanosoma cruzi* and *Leishmania* interaction with their host (s): Exploitation of immune and defense mechanisms by the parasite leading to persistence and chronicity, features reminiscent of immune system evasion strategies in cancer diseases. Arch. Immunol. Ther. Exp..

[99] Kedzierski L., Montgomery J., Bullen D., Curtis J., Gardiner E., Jimenez-Ruiz A., Handman E. (2004). A leucine-rich repeat motif of *Leishmania* parasite surface antigen 2 binds to macrophages through the complement receptor 3. J. Immunol..

[100] Pereira Filho A.A., de Sousa Nascimento A.A., Saab N.A.A., Fugiwara R.T., Pessoa G.C.D.Á., Koerich L.B., Pereira M.H., Araújo R.N., Sant’Anna M.R.V., Gontijo N.F. (2021). Evasion of the complement system by *Leishmania* through the uptake of factor H, a complement regulatory protein. Acta Trop..

[101] Kim Y.U., Hong Y. (2007). Functional analysis of the first mannosyltransferase (PIG-M) involved in glycosylphosphatidylinositol synthesis in *Plasmodium falciparum*. Mol. Cells.

[102] Reyes A.C., Encina J.L.R. (2019). Trypanosoma cruzi infection: Mechanisms of evasion of immune response. Biology of Trypanosoma cruzi.

[103] Cestari I., Ansa-Addo E., Deolindo P., Inal J.M., Ramirez M.I. (2012). *Trypanosoma cruzi* immune evasion mediated by host cell-derived microvesicles. J. Immunol..

[104] De Castro Neto A.L., Da Silveira J.F., Mortara R.A. (2021). Comparative analysis of virulence mechanisms of trypanosomatids pathogenic to humans. Front. Cell. Infect. Microbiol..

[105] Sosoniuk E., Vallejos G., Kenawy H., Gaboriaud C., Thielens N., Fujita T., Schwaeble W., Ferreira A., Valck C. (2014). *Trypanosoma cruzi* calreticulin inhibits the complement lectin pathway activation by direct interaction with L-Ficolin. Mol. Immunol..

[106] Brittingham A., Morrison C.J., McMaster W.R., McGwire B.S., Chang K.-P., Mosser D.M. (1995). Role of the *Leishmania* surface protease gp63 in complement fixation, cell adhesion, and resistance to complement-mediated lysis. J. Immunol..

[107] Chaudhuri G., Chang K.-P. (1988). Acid protease activity of a major surface membrane glycoprotein (gp63) from *Leishmania mexicana* promastigotes. Mol. Biochem. Parasitol..

[108] Nunes A., Almeida-Campos F., Horta M., Ramalho-Pinto F. (1997). *Leishmania amazonensis* promastigotes evade complement killing by interfering with the late steps of the cascade. Parasitology.

[109] Verma S., Mandal A., Ansari M.Y., Kumar A., Abhishek K., Ghosh A.K., Kumar A., Kumar V., Das S., Das P. (2018). *Leishmania donovani* inhibitor of serine peptidases 2 mediated inhibition of lectin pathway and upregulation of C5aR signaling promote parasite survival inside host. Front. Immunol..

[110] Lima P.C., Mottram J.C. (2010). Trypanosomatid-encoded inhibitors of peptidases: Unique structural features and possible roles as virulence factors. Open Parasitol. J..

[111] Lamotte S., Späth G.F., Rachidi N., Prina E. (2017). The enemy within: Targeting host–parasite interaction for antileishmanial drug discovery. PLoS Negl. Trop. Dis..

[112] Miller H.W., Tam T.S., Ralston K.S. (2022). *Entamoeba histolytica* Develops Resistance to Complement Deposition and Lysis after Acquisition of Human Complement-Regulatory Proteins through Trogocytosis. Mbio.

[113] Faria C.P., Neves B.M., Lourenço Á., Cruz M.T., Martins J.D., Silva A., Pereira S., Sousa M.d.C. (2020). *Giardia lamblia* decreases NF-κB p65RelA protein levels and modulates LPS-induced pro-inflammatory response in macrophages. Sci. Rep..

[114] Ibáñez-Escribano A., Nogal-Ruiz J.J., Pérez-Serrano J., Gómez-Barrio A., Escario J.A., Alderete J. (2015). Sequestration of host-CD59 as potential immune evasion strategy of *Trichomonas vaginalis*. Acta Trop..

[115] Kurtovic L., Behet M.C., Feng G., Reiling L., Chelimo K., Dent A.E., Mueller I., Kazura J.W., Sauerwein R.W., Fowkes F.J. (2018). Human antibodies activate complement against *Plasmodium falciparum* sporozoites, and are associated with protection against malaria in children. BMC Med..

[116] Boyle M., Chan J., Handayuni I., Reiling L., Feng G., Hilton A., Kurtovic L., Oyong D., Piera K., Barber B. (2019). IgM in human immunity to *Plasmodium falciparum* malaria. Sci. Adv..

[117] Behet M.C., Kurtovic L., van Gemert G.-J., Haukes C.M., Siebelink-Stoter R., Graumans W., van de Vegte-Bolmer M.G., Scholzen A., Langereis J.D., Diavatopoulos D.A. (2018). The complement system contributes to functional antibody-mediated responses induced by immunization with *Plasmodium falciparum* malaria sporozoites. Infect. Immun..

[118] Reiling L., Boyle M.J., White M.T., Wilson D.W., Feng G., Weaver R., Opi D.H., Persson K.E., Richards J.S., Siba P.M. (2019). Targets of complement-fixing antibodies in protective immunity against malaria in children. Nat. Commun..

[119] Ubillos I., Ayestaran A., Nhabomba A.J., Dosoo D., Vidal M., Jiménez A., Jairoce C., Sanz H., Aguilar R., Williams N.A. (2018). Baseline exposure, antibody subclass, and hepatitis B response differentially affect malaria protective immunity following RTS, S/AS01E vaccination in African children. BMC Med..

[120] Zenklusen I., Jongo S., Abdulla S., Ramadhani K., Lee Sim B.K., Cardamone H., Flannery E.L., Nguyen T., Fishbaugher M., Steel R.W. (2018). Immunization of malaria-preexposed volunteers with PfSPZ vaccine elicits long-lived IgM invasion-inhibitory and complement-fixing antibodies. J. Infect. Dis..

[121] Autheman D., Crosnier C., Clare S., Goulding D.A., Brandt C., Harcourt K., Tolley C., Galaway F., Khushu M., Ong H. (2021). An invariant *Trypanosoma vivax* vaccine antigen induces protective immunity. Nature.

[122] Gruszynski A.E., van Deursen F.J., Albareda M.C., Best A., Chaudhary K., Cliffe L.J., del Rio L., Dunn J.D., Ellis L., Evans K.J. (2006). Regulation of surface coat exchange by differentiating African trypanosomes. Mol. Biochem. Parasitol..

[123] Moreno C.J.G., Temporão A., Torres T., Sousa Silva M. (2019). *Trypanosoma brucei* interaction with host: Mechanism of VSG release as target for drug discovery for african trypanosomiasis. Int. J. Mol. Sci..

[124] Ferreira V.P., Pangburn M.K., Cortés C. (2010). Complement control protein factor H: The good, the bad, and the inadequate. Mol. Immunol..

[125] Van Beek A.E., Sarr I., Correa S., Nwakanma D., Brouwer M.C., Wouters D., Secka F., Anderson S.T., Conway D.J., Walther M. (2018). Complement factor H levels associate with *Plasmodium falciparum* malaria susceptibility and severity. Open Forum Infect. Dis..

[126] Macleod O.J., Bart J.-M., MacGregor P., Peacock L., Savill N.J., Hester S., Ravel S., Sunter J.D., Trevor C., Rust S. (2020). A receptor for the complement regulator factor H increases transmission of trypanosomes to tsetse flies. Nat. Commun..

[127] Mulamba C., Williams C., Kreppel K., Ouedraogo J.B., Olotu A.I. (2022). Evaluation of the Pfs25-IMX313/Matrix-M malaria transmission-blocking candidate vaccine in endemic settings. Malar. J..

[128] Kipnis T.L., David J.R., Alper C.A., Sher A., Da Silva W.D. (1981). Enzymatic treatment transforms trypomastigotes of *Trypanosoma cruzi* into activators of alternative complement pathway and potentiates their uptake by macrophages. Proc. Natl. Acad. Sci. USA.

[129] Giuliani M.M., Adu-Bobie J., Comanducci M., Aricò B., Savino S., Santini L., Brunelli B., Bambini S., Biolchi A., Capecchi B. (2006). A universal vaccine for serogroup B meningococcus. Proc. Natl. Acad. Sci. USA.

[130] Orsini F., De Blasio D., Zangari R., Zanier E.R., De Simoni M.-G. (2014). Versatility of the complement system in neuroinflammation, neurodegeneration and brain homeostasis. Front. Cell. Neurosci..

[131] Sultan B.A., AL-Fatlawi S.N. (2016). Assessment of C3 and C4 component of complement system in aborted women infected with *Toxoplasma gondii*. Al-Qadisiyah Med. J..

[132] Leite P.E.C., de Araujo Portes J., Pereira M.R., Russo F.B., Martins-Duarte E.S., Dos Santos N.A., Attias M., Barrantes F.J., Beltrão-Braga P.C.B., de Souza W. (2021). Morphological and biochemical repercussions of *Toxoplasma gondii* infection in a 3D human brain neurospheres model. Brain Behav. Immun. Health.

[133] Yang H.-Y., Chien W.-C., Chung C.-H., Su R.-Y., Lai C.-Y., Yang C.-C., Tzeng N.-S. (2021). Risk of dementia in patients with toxoplasmosis: A nationwide, population-based cohort study in Taiwan. Parasit. Vectors.

[134] Nayeri T., Sarvi S., Sharif M., Daryani A. (2021). *Toxoplasma gondii*: A possible etiologic agent for Alzheimer’s disease. Heliyon.

[135] de Bles N.J., van der Does J.E., Kortbeek L.M., Hofhuis A., van Grootheest G., Vollaard A.M., Schoevers R.A., van Hemert A.M., Penninx B.W., Rius-Ottenheim N. (2021). *Toxoplasma gondii* seropositivity in patients with depressive and anxiety disorders. Brain Behav. Immun. Health.

[136] Roe K. (2021). The link between *Toxoplasma gondii* infections and higher mortality in COVID-19 patients having schizophrenia. Eur. Arch. Psychiatry Clin. Neurosci..

[137] Lv L., Wang Y., Feng W., Hernandez J.A., Huang W., Zheng Y., Zhou X., Lv S., Chen Y., Yuan Z.-G. (2017). iTRAQ-based differential proteomic analysis in Mongolian gerbil brains chronically infected with *Toxoplasma gondii*. J. Proteom..

[138] Ziabska K., Ziemka-Nalecz M., Pawelec P., Sypecka J., Zalewska T. (2021). Aberrant Complement System Activation in Neurological Disorders. Int. J. Mol. Sci..

[139] Sekar A., Bialas A.R., De Rivera H., Davis A., Hammond T.R., Kamitaki N., Tooley K., Presumey J., Baum M., Van Doren V. (2016). Schizophrenia risk from complex variation of complement component 4. Nature.

[140] Sekar A., Bialas A.R., de Rivera H., Davis A., Hammond T.R., Kamitaki N., Tooley K., Presumey J., Baum M., Van Doren V. (2022). Author Correction: Schizophrenia risk from complex variation of complement component 4. Nature.

[141] Rachidi N., Taly J.F., Durieu E., Leclercq O., Aulner N., Prina E., Pescher P., Notredame C., Meijer L., Späth G.F. (2014). Pharmacological assessment defines *Leishmania donovani* casein kinase 1 as a drug target and reveals important functions in parasite viability and intracellular infection. Antimicrob. Agents Chemother..

[142] Ishino T., Yano K., Chinzei Y., Yuda M., Ward G. (2004). Cell-passage activity is required for the malarial parasite to cross the liver sinusoidal cell layer. PLoS Biol..

[143] Ishino T., Chinzei Y., Yuda M. (2005). A *Plasmodium* sporozoite protein with a membrane attack complex domain is required for breaching the liver sinusoidal cell layer prior to hepatocyte infection. Cell. Microbiol..

[144] Hamaoka B.Y., Ghosh P. (2014). Structure of the essential *Plasmodium* host cell traversal protein SPECT1. PLoS ONE.

[145] Sharma S., Kumar G., Vashishta M., Pandey R., Rathore S., Chourasia B.K., Singhal J., Deshmukh A., Kalamuddin M., Paul G. (2018). Biochemical characterization of *Plasmodium* complement factors binding protein for its role in immune modulation. Biochem. J..

[146] Sijwali P.S. (2018). Interaction with complement proteins and dendritic cells implicates LCCL domain-containing proteins (CCps) of malaria parasites in immunomodulation. Biochem. J..

[147] Torrecilhas A.C., Schumacher R.I., Alves M.J.M., Colli W. (2012). Vesicles as carriers of virulence factors in parasitic protozoan diseases. Microbes Infect..

[148] Wilson J.G., Murphy E.E., Wong W.W., Klickstein L., Weis J.H., Fearon D.T. (1986). Identification of a restriction fragment length polymorphism by a CR1 cDNA that correlates with the number of CR1 on erythrocytes. J. Exp. Med..

[149] Opi D.H., Swann O., Macharia A., Uyoga S., Band G., Ndila C.M., Harrison E.M., Thera M.A., Kone A.K., Diallo D.A. (2018). Two complement receptor one alleles have opposing associations with cerebral malaria and interact with α^+^thalassaemia. Elife.

[150] Beaudoin C.A., Jamasb A.R., Alsulami A.F., Copoiu L., van Tonder A.J., Hala S., Bannerman B.P., Thomas S.E., Vedithi S.C., Torres P.H. (2021). Predicted structural mimicry of spike receptor-binding motifs from highly pathogenic human coronaviruses. Comput. Struct. Biotechnol. J..

[151] Wong S.S.W., Daniel I., Gangneux J.-P., Jayapal J.M., Guegan H., Dellière S., Lalitha P., Shende R., Madan T., Bayry J. (2020). Differential interactions of serum and bronchoalveolar lavage fluid complement proteins with conidia of airborne fungal pathogen *Aspergillus fumigatus*. Infect. Immun..

[152] Bayer-Santos E., Aguilar-Bonavides C., Rodrigues S.P., Cordero E.M., Marques A.F., Varela-Ramirez A., Choi H., Yoshida N., Da Silveira J.F., Almeida I.C. (2013). Proteomic analysis of *Trypanosoma cruzi* secretome: Characterization of two populations of extracellular vesicles and soluble proteins. J. Proteome Res..

[153] Rashidi S., Mojtahedi Z., Shahriari B., Kalantar K., Ghalamfarsa G., Mohebali M., Hatam G. (2019). An immunoproteomic approach to identifying immunoreactive proteins in *Leishmania infantum* amastigotes using sera of dogs infected with canine visceral leishmaniasis. Pathog. Glob. Health.

[154] Butterworth S., Torelli F., Lockyer E.J., Wagener J., Song O.-R., Broncel M., Russell M., Young J.C., Treeck M. (2022). *Toxoplasma gondii* ROP1 subverts murine and human innate immune restriction. bioRxiv.

[155] Nguyen V.A., Riddell N., Crewther S.G., Faou P., Rajapaksha H., Howells D.W., Hankey G.J., Wijeratne T., Ma H., Davis S. (2020). Longitudinal Stroke Recovery Associated With Dysregulation of Complement System—A Proteomics Pathway Analysis. Front. Neurol..

